# A Successful Pregnancy in a Hemihysterectomized Patient–A Case Report

**DOI:** 10.1155/2022/4627241

**Published:** 2022-12-12

**Authors:** Sofia Jovina Domingues, Luísa Pinto

**Affiliations:** ^1^Centro Hospitalar de Setúbal, Setúbal, Portugal; ^2^Centro Hospitalar e Universitário Lisboa Norte, Lisbon, Portugal

## Abstract

**Introduction:**

Didelphys uterus, two separate uterine horns or a double uterus with two separate cervices, is one of several congenital uterine anomalies (CUA), accounting for 5% of CUA. CUA could be associated with dysmenorrhea, pelvic pain, and decreased fertility. During pregnancy, it has been associated with higher risk of miscarriage, preterm birth, malpresentation, and fetal growth restriction. There still is insufficient evidence on the efficacy and safety of surgical interventions in CUA, including hemihysterectomy, in what improvement of reproductive performance is concerned.

**Objective:**

The aim of the present case report is to review the literature and complement information on pregnancy outcomes after hemihysterectomy, contributing to a better counseling of women planning a pregnancy under these circumstances. *Case Presentation*. This paper case reports a successful pregnancy in a woman previously submitted to a hemihysterectomy and removal of a vaginal septum by hysteroscopy, due to a symptomatic didelphys uterus. An ipsilateral renal agenesis was also present. A cesarean was performed at 38 weeks' gestation and a healthy baby was born. *Discussion.* This paper reports an exceptional clinical situation, with only 9 cases described in the last 6 decades. As in previously reported cases, a cesarean was performed at term, resulting in a live childbirth. In these cases, a higher live birth rate and a lower incidence of preterm deliveries was found, comparing with uterine didelphys pregnancies.

**Conclusion:**

Understanding both the exact nature of a uterine anomaly before a surgical treatment and the prognosis for a future pregnancy after the intervention are of paramount importance for precise counseling of future parents.

## 1. Introduction

Congenital uterine anomalies (CUA) resulted from abnormal formation, differentiation, and/or fusion of the Müllerian or paramesonephric ducts during fetal life [1]. Coexisting renal, ureteral, and bladder anomalies are present in up to 40% of these patients [2]. There is no universally accepted classification system for CUA, but it seems that the classifications from the American Fertility Society (AFS) and from the European Society of Human Reproduction and Embryology/European Society for Gynecological Endoscopy (ESHRE/ESGE) have been the most widely accepted [3]. The prevalence of CUA in the general population varies from 0.06 to 38%. Most CUA are asymptomatic but they can be associated with adverse pregnancy outcomes [3]. Using AFS classification, didelphys uterus accounts for 5% of CUA and results from the lack of fusion of the paired Mullerian ducts, which should occur between the 6^th^ and the 11^th^ week of gestation [1]. The phenotype is that of two separate uterine horns or a double uterus with two separate cervices. Each uterine horn is linked to one fallopian tube and ovary [4]. A longitudinal vaginal septum is present in 75% of cases [1]. There still is insufficient evidence on the efficacy and safety of surgical interventions in CUA, in what improvement of reproductive performance is concerned. Surgical correction of CUA should be decided on an individual basis, depending on clinical symptoms, type of anomaly, and reproductive project.

We report a successful pregnancy in a woman previously submitted to surgical treatment for a didelphys uterus and a right-obstructed hemivagina (hemihysterectomy and hysteroscopic removal of a vaginal septum). There are only 9 cases in the literature describing pregnancy outcomes after a hemihysterectomy. The main purpose of this case report is to review the literature and complement information on pregnancy outcomes after hemihysterectomy and consequently contribute to a better counseling of women/couples planning a pregnancy under these circumstances. Patient consent for this report was obtained.

## 2. Case Presentation

A 25-year-old woman was referred to our maternal-fetal outpatient clinic at 20 weeks' gestation, due to a clinical history of CUA submitted to a surgical intervention 11 years before. Menarche occurred at the age of 11 and since then, she complained of severe dysmenorrhea, pelvic pain, and heavy menstrual bleeding. Pain did not decrease with the use of estroprogestative pill, anti-inflammatory drugs, or other pain killers. At 13 years of age, a gynecological appointment revealed normal stature and normal external genitalia, but gynecological examination was hampered by the presence of an intact hymen. Additional investigation with abdominal ultrasound showed two separate uterine cavities, and a single left kidney. Based on the suspicion of a CUA, at the age of 14, the patient underwent a diagnostic laparoscopy–which revealed a double uterus–and a diagnostic hysteroscopy which identified a longitudinal vaginal septum and a right-obstructed hemivagina. The laparoscopic approach was converted to open surgery and a supracervical right hemihysterectomy was performed. At the same operative time a hysteroscopic resection of the vaginal septum was undertaken and two uterine cervices were visualized. One year later, an abdominal ultrasound revealed an anteverted uterus with mild left laterality and normal dimensions (98 × 26 × 45 mm, longitudinal, anteroposterior, and transversal axes). At the age of 17 she initiated sexual life with no complaints and 5 years later she got pregnant and had a spontaneous abortion at 7 weeks' gestation for which no surgical treatment was needed.

Regarding current pregnancy, no fetal or maternal relevant complications occurred. Pregnancy surveillance was done according to department protocols, including obstetrical ultrasounds in every trimester of gestation. Route of delivery was discussed with the patient and given the previous uterine surgery, an elective cesarean was scheduled to week 39. However, gestational hypertension at 38 weeks' gestation led to the decision to terminate the pregnancy. An uneventful cesarean resulted in a male newborn, 3170 g, Apgar index 9/10 at 1 and 5 minutes, respectively. Immediately after the cesarean, normal blood pressure was restored and no other meaningful clinical events occurred.

## 3. Discussion

We describe a successful pregnancy in a woman previously submitted to a right hemihysterectomy due to the diagnosis of didelphys uterus.

The precise incidence of CUA in the general population is not known since most CUA are asymptomatic, they are diagnosed by different techniques and classified using different classification systems [3]. Accurate evaluation of the internal and external contours of the uterus is crucial in making the diagnosis and classifying CUA correctly. In the past, as reported in our case, the gold standard was a combination of laparoscopy and hysteroscopy. Currently, 3D transvaginal sonography (TVS) is considered the gold standard for the assessment of CUA, correctly classifying the different types of uterine anomalies [4]. Magnetic resonance imaging (MRI) of the pelvis has a high sensitivity and specificity for the diagnosis of CUA and is helpful in delineating the endometrium, detecting uterine horns and also in defining renal anatomy. MRI is not routinely recommended in women with a suspected CUA but it seems useful for those with unconfirmed diagnosis on 3D ultrasound, with suspected complex anomalies or who decline or are distressed by internal examinations [4].

Didelphys uterus can be suspected when two endometrial cavities are visualized in the transverse plane of conventional 2D ultrasound and 3D ultrasound, associated with a clinical demonstration of two cervices and/or two vaginas on speculum examination, can confirm the diagnosis [4].

CUA may be associated with congenital renal anomalies, mostly unilateral renal agenesis, as described in this paper, because of their closely related embryonic origin. Therefore, a urinary tract ultrasound, an MRI, or an intravenous pyelogram should be recommended [5].

CUA can be associated with adverse pregnancy outcomes. A recent systematic review and meta-analysis showed that didelphys uterus was not associated with an increased incidence of infertility or miscarriage, but it was mainly associated with a higher risk of preterm delivery, preterm premature rupture of membranes, fetal malpresentation, cesarean, fetal growth restriction, and small for gestational age [3]. Regarding the higher risk of cesarean in this CUA, Cwiertnia et al. demonstrated that the decision to perform cesarean depends on several factors, including fetal presentation, patient preference, and vaginal septum features [6].

However, there is a lack of evidence on improved reproductive outcomes after surgical interventions for CUA. The goal of CUA management is to correct anatomical distortions associated with obstructive anomalies in order to relieve symptoms, thereby improving quality of life, and to avoid long-term health and reproductive adverse consequences. In nonobstructive anomalies, the objective is to improve reproductive outcomes in infertile women or in women who have experienced recurrent miscarriages [4].

In our report, there was a suspicion of CUA on abdominal ultrasound (TVS was not performed due to the presence of an intact hymen) and then a hysteroscopy and a laparoscopy were performed. Given the severe pain, suggesting an obstructive anomaly, a hemihysterectomy and removal of a vaginal septum were performed, aiming to improve the quality of life. After surgical treatment, the symptoms are resolved.

Nine cases of pregnant women after hemihysterectomy have been reported since 1947 (Table 1). In these cases, most clinicians decided for a cesarean section, 82% of pregnancies resulted in live births and 37% in preterm deliveries.

A previous study including 114 pregnant patients with didelphys uterus showed a 56% live birth rate and 43% preterm delivery rate [13]. More recently, preterm delivery rates in women with didelphys uterus have been described as higher than post-hemihysterectomy surgery (66.7% vs. 44.4%) [14].

Although the reduced number of pregnancies after hemihysterectomy reported in literature, our case adds and corroborates information about pregnancy management after a hemihysterectomy, showing an adequate live birth. Regarding to preterm delivery rate, it should be interpreted individually with caution, because there are several factors to contribute it, such as a multiple pregnancy.

The strength of our case report relies on accurate description of hemihysterectomy and pregnancy outcomes after surgery, contributing to our knowledge on this subject. Prospective long-term studies in hemihysterectomized women should be carried out, to better assess pregnancy outcomes.

## 4. Conclusion

The decision to perform a surgery, such as a hemihysterectomy, at a young stage of life in order to improve quality of life must be considered, taking into account the risks and benefits, in the short and long term.

Understanding the exact nature of the uterine anomaly before a surgical treatment and explaining to the patient the prognosis of a future pregnancy is of paramount importance for precise counseling and adequacy of expectations. We report the case of a successful pregnancy after a hemihysterectomy; however, it is important to be aware of the possible different clinical implication for specific types of uterine anomalies and surgeries.

## Figures and Tables

**Table 1:** Pregnancy outcomes after hemihysterectomy (adapted from Kai et al. [7]).

Author (year)	Patient (*n*)	Pregnancies	Abortions	Preterm delivery	Term delivery	Type of delivery	Live births	Uterine rupture
Smith et al. [8]	1	1	0	0	1	Cesarean	1	0
Thelwall-Jone (1976) [9]	2	3	0	1	2	Cesarean	3^a^	0
Phillips [10]	1	1	1	0	0	Cesarean	0	0
Candiani et al. [11]	2	2	1	1	0	Cesarean	1	0
Berhan et al. [12]	1	1	0	0	1	Vaginal	1	0
Kai et al. [7]	1	2	0	2	0	Cesarean	2	0
Present case	1	1	0	0	1	Cesarean	1	0
TOTAL	9	11	2	4	4	—	9	0

^a^ One singleton pregnancy and one twin pregnancy.

## Data Availability

All data used to support the findings of this study are included within the article.
